# Upadacitinib sustained-release tablets for the treatment of chronic refractory gouty arthritis: a case report and literature review

**DOI:** 10.3389/fmed.2024.1357117

**Published:** 2024-03-28

**Authors:** Lishi Yu, Weidong Huang, Honghua Lv, Lie Jin, Wenhui Lei

**Affiliations:** ^1^Department of Rheumatology, The Fifth Affiliated Hospital of Wenzhou Medical University, Lishui, China; ^2^Department of Nephrology, The Fifth Affiliated Hospital of Wenzhou Medical University, Lishui, China

**Keywords:** gout, arthritis, gouty arthritis, treatment, upadacitinib sustained-release tablets

## Abstract

**Background:**

Gouty arthritis (GA) is a crystal-related joint disease caused by the deposition of monosodium urate (MSU) crystals, directly associated with hyperuricemia resulting from purine metabolism disorder and/or reduced uric acid excretion. Acute attacks of typical gouty arthritis are generally relieved through the clinical use of NSAIDs, colchicine, or glucocorticoids. However, managing patients with chronic refractory gout poses challenges due to complications such as multiple tophi, gouty nephropathy, diabetes, and gastrointestinal bleeding. While there have been numerous studies on gout in recent years, research specifically regarding chronic refractory gout remains limited. The management of such cases still faces several unresolved issues, including recurrent disease flare-ups and poor patient compliance leading to inadequate drug utilization and increased risk of side effects. In this report, we present a case of successful improvement in chronic refractory gouty arthritis using the biologic agent upadacitinib sustained-release tablets.

**Case presentation:**

Our case report involves a 53 years-old Asian patient with recurrent gouty arthritis who had a history of over 20 years without regular treatment, presenting with tophi and an increasing number of painful episodes. During hospitalization, various analgesics and anti-inflammatory drugs provided inadequate relief, requiring the use of steroids to alleviate symptoms. However, tapering off steroids proved challenging. We decided to add upadacitinib sustained-release tablets to the treatment regimen, which ultimately improved the patient’s condition. After 6 months of follow-up, the patient has not experienced any further acute pain episodes.

**Conclusion:**

This case highlights the potential therapeutic effect of upadacitinib sustained-release tablets during the acute phase of chronic refractory gouty arthritis.

## Introduction

Gouty arthritis (GA) is characterized by recurrent attacks of redness, swelling, heat, pain, and functional impairment in the affected joints. As the disease progresses, symptoms worsen, leading not only to joint deformities but also potential kidney damage and the onset of cardiovascular diseases, significantly impacting the physical and mental health as well as the quality of life of patients. With the improvement in living standards, there has been a significant increase in the intake of proteins, fats, and sugars. In the United States, the current prevalence of gout has reached 3.9%, showing an upward trend annually (1). Clinical management of gout arthritis typically involves physical therapy, pharmacological treatment, and surgical interventions. Due to the complex pathogenesis and diverse etiology of gout arthritis, there is currently no definitive cure. Clinically, acute gout attacks are primarily alleviated with medications such as colchicine, nonsteroidal anti-inflammatory drugs, and corticosteroids, along with uric acid synthesis inhibitors like allopurinol and uricosuric agents such as probenecid to lower uric acid levels. However, the use of these medications is limited in clinical practice due to risks such as gastrointestinal reactions, allergic rashes, and potential harm to liver and kidney function.

The Janus kinase-signal transducer and activator of transcription (JAK-STAT) pathway plays a crucial role in the pathogenesis and progression of rheumatic immune diseases. Since the advent of JAK inhibitors, their applications in the field of rheumatic immunology have become increasingly widespread. Currently, they are recommended for the treatment of various rheumatic immune diseases, including rheumatoid arthritis, psoriatic arthritis, ankylosing spondylitis, juvenile idiopathic arthritis, among others. Upadacitinib is a novel selective JAK inhibitor that exhibits increased selectivity for JAK1. In recent years, it has been extensively studied in multiple disease fields, particularly in rheumatoid arthritis. However, to our knowledge, its use in the treatment of gouty arthritis has not been documented. In this report, we present a case of refractory gouty arthritis successfully treated with upadacitinib sustained-release tablets.

## Case presentation

A 53 years-old Asian male, a civil servant by profession, with a history of smoking and drinking, experienced joint pain in his youth, which was diagnosed as gouty arthritis. Initially, the duration of pain during flare-ups was short, and the analgesics provided relief, so he did not pay much attention to high uric acid levels. His highest recorded uric acid level was over 800+. Over time, the frequency and intensity of his joint pain episodes increased. Due to his busy work schedule, the patient relied on painkillers as needed and occasionally used allopurinol for uric acid-lowering treatment.

Subsequently, the patient developed arthritis in the knee joints and hand joints, with the appearance of tophi in the joints approximately 10 years ago. During this period, he received on-demand treatment with colchicine, febuxostat, and diclofenac sodium. Starting this year, he experienced flare-ups every 2–3 months. Half a month prior to his presentation at our hospital, the patient had another episode of severe joint pain affecting the knees, ankles, and hand joints, accompanied by limited mobility. He self-medicated with diclofenac sodium and colchicine tablets, but the effect was unsatisfactory. Consequently, he sought medical attention and was treated with intravenous dexamethasone 7.5 mg, which resulted in reduced pain. The attending physician recommended hospitalization for further treatment. The main laboratory results are shown in Table 1.

**Table 1:** Display of main laboratory results and normal range upon admission, March 2023.

The laboratory parameters	Results
White blood cells	7.8 × 109/L (3.5–9.5)
Haemoglobin	134 g/L (130–175)
Erythrocyte	4.21 × 1,012/L (4.3–5.8)
ESR	55 mm/1 h (0–15)
CRP	53.24 mg/L (<8)
Albumin	39.6 g/L (40–55)
Creatinine	84 μmol/L (57–97)
Total cholesterol	3.26 mmol/L (<5.2)
Triglycerides	1.08 mmol/L (<1.71)
Glutamyl transpeptidase	328 U/L (10–60)
Uric acid	555 μmol/L (208–428)
Serum ferritin	732.2 ng/mL (21.8–274.2)

ESR, erythrocyte sedimentation rate; CRP, serum C-reactive protein.

**Figure 1:**
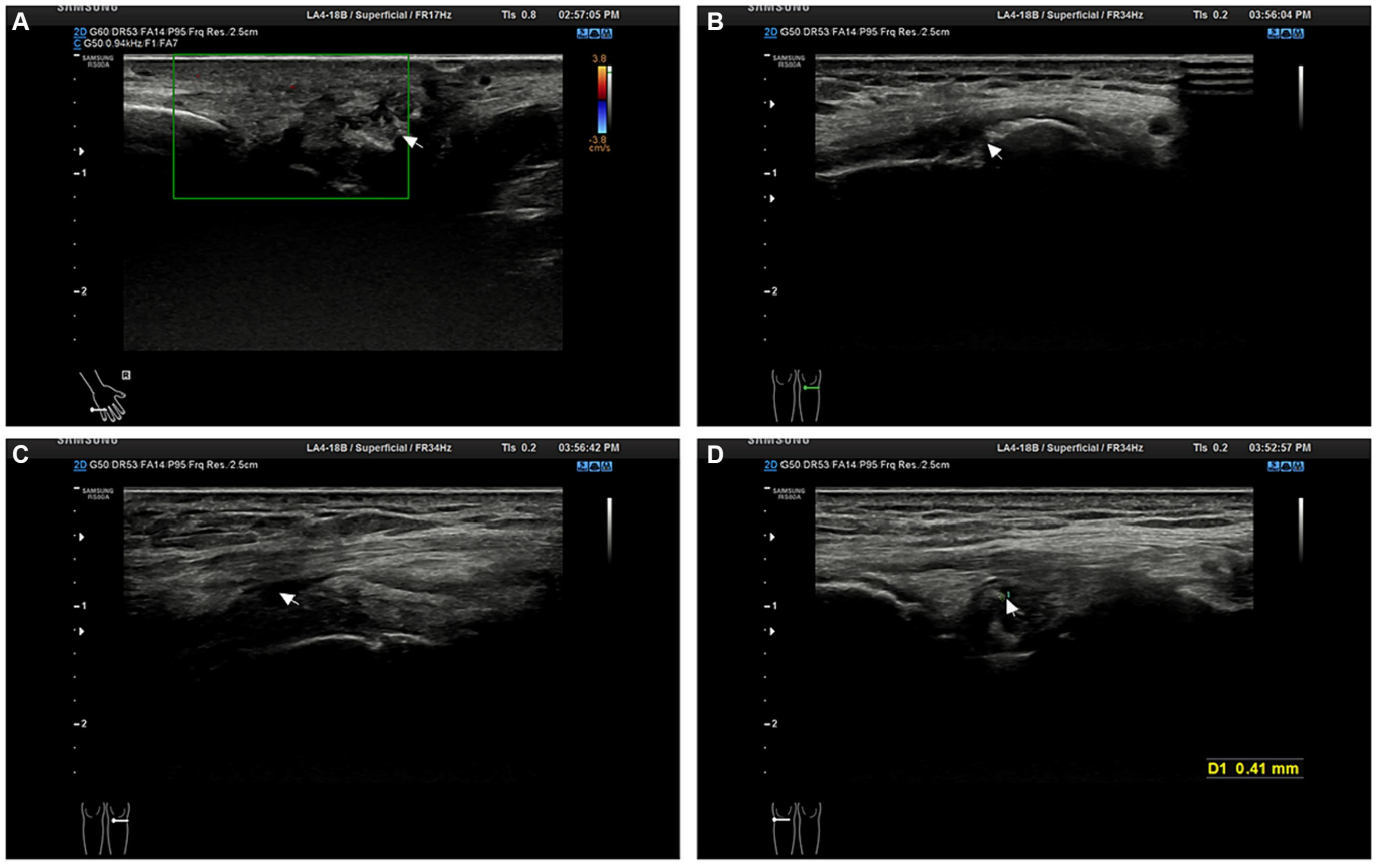
Patient joint ultrasonography images. **(A)** The image shows synovial proliferation with bone erosion in the interphalangeal joints of the right hand. **(B)** The image suggests bone erosion in the knee joint. **(C)** The image reveals knee joint effusion. **(D)** The image demonstrates the formation of tophi (size *D* = 0.41 mm, indicated by the arrow).

**Figure 2:**
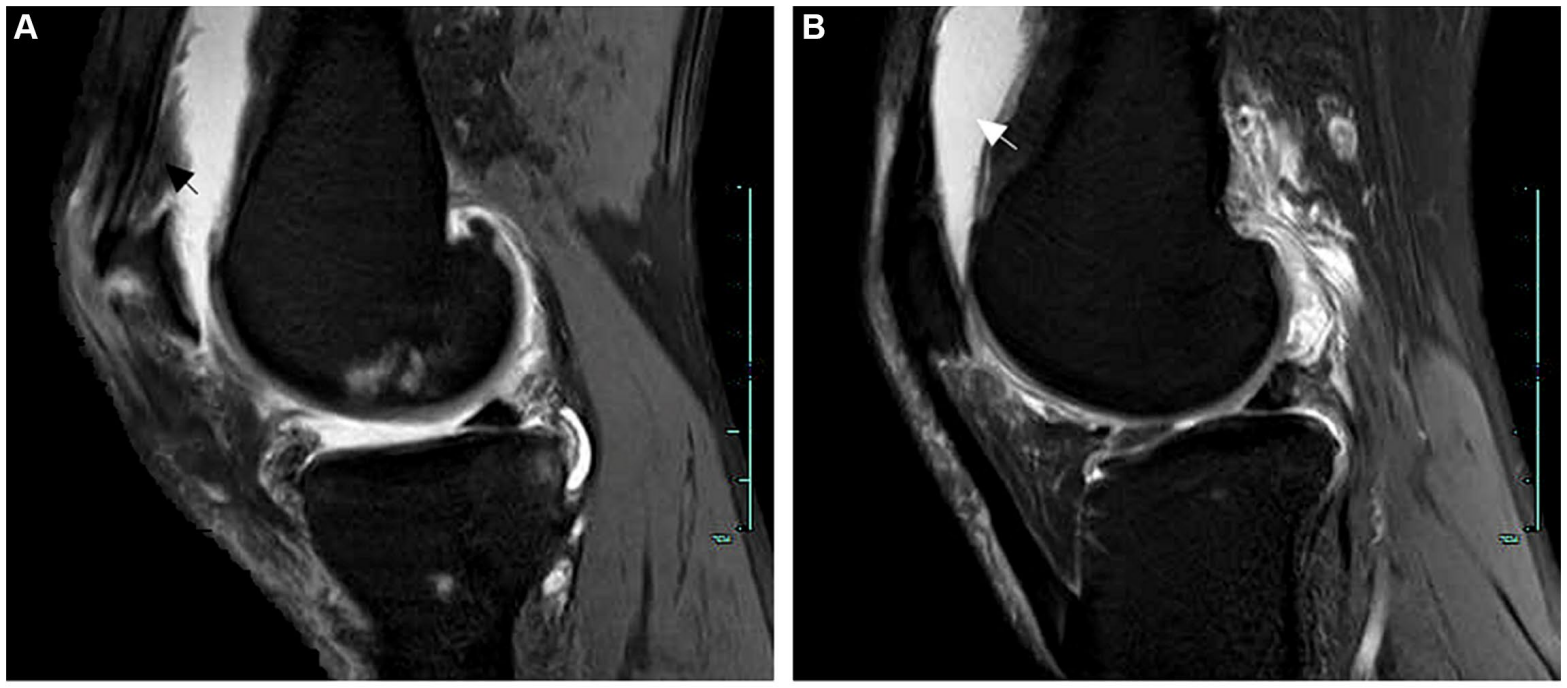
MRI examination of the right knee joint. **(A)** The arrow indicates synovitis. **(B)** The arrow points towards joint cavity effusion.

Upon admission, physical examination revealed tophi in the first metatarsophalangeal joint of the left foot and the small finger of the right hand. Multiple joints were tender, and there was limited range of motion. Laboratory investigations are detailed in Table 1. Electrocardiography showed sinus rhythm and a Q wave in lead III and aVF. Echocardiography indicated left ventricular hypertrophy. Abdominal ultrasound revealed fatty liver, gallbladder polyps, and a cyst in the right kidney. Further imaging studies were conducted to evaluate the affected joints. Hand joint ultrasonography revealed possible synovial hyperplasia with tophi formation in the bilateral first, second, and fifth metacarpophalangeal joints, and erosions in the first and fifth metacarpophalangeal joints of the right hand. Knee joint ultrasonography showed synovial hyperplasia with erosions in both knee joints, as well as joint effusion in the right knee. Tophi formation was also possible in the knee joints (Figure 1). Plain radiography of the right knee showed degenerative changes, synovitis, joint effusion, posterior cruciate ligament injury, and degeneration of the medial and lateral menisci (Figure 2).

According to the patient’s clinical manifestations and relevant examinations, and based on the ACR/EULAR 2015 diagnostic criteria for gout, other rheumatic systemic diseases were ruled out. The patient scored above 8 points, confirming the diagnosis of gouty arthritis. After admission, despite the use of colchicine and low-dose steroids, his pain did not improve. Therefore, on the second day of hospitalization, we administered intravenous methylprednisolone 20 mg daily for 5 days, combined with tumor necrosis factor-alpha (TNF-α) antagonists and intra-articular steroid injections. The patient reported subjective improvement and was discharged. He was prescribed oral prednisone 5 mg three times a day upon discharge.

**Figure 3:**
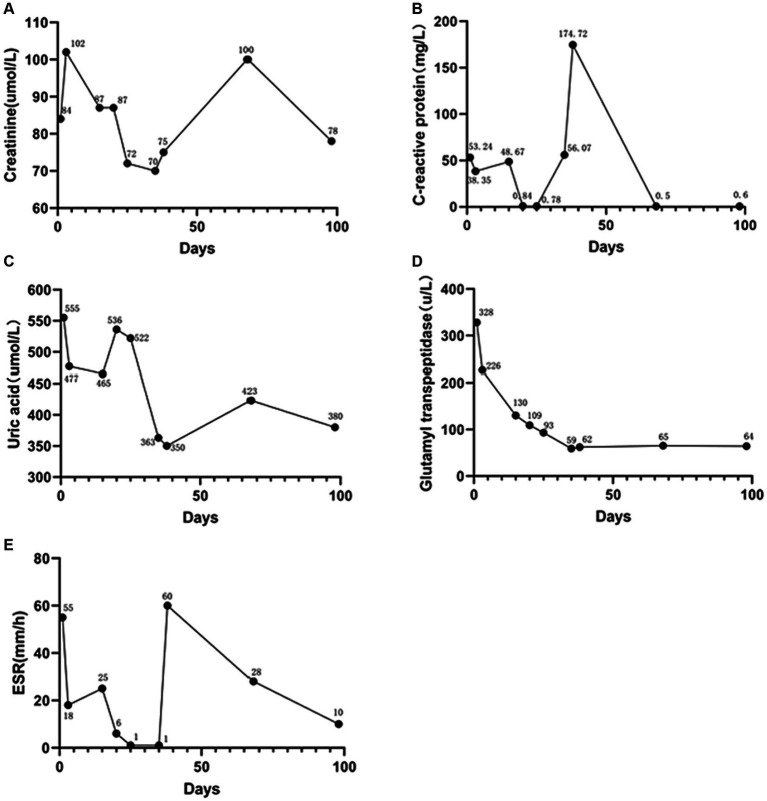
Patient post-treatment laboratory parameter change curves. **(A)** Serum creatinine. **(B)** C-reactive protein. **(C)** Serum uric acid. **(D)** Glutamyl transpeptidase. **(E)** Erythrocyte sedimentation rate. ESR, erythrocyte sedimentation rate.

Ten days later, he experienced generalized joint pain again. We increased the steroid dosage to intravenous methylprednisolone 40 mg daily for 6 days, in combination with tocilizumab injection (an IL-6 inhibitor). Unfortunately, the treatment did not yield satisfactory results, and the patient still complained of joint pain, requiring further escalation of steroid dosage. At this point, we decided to add upadacitinib tablets. Thankfully, the steroid dosage was eventually successfully reduced. The patient was discharged 10 days later (prescribed prednisone 10 mg three times a day), and 1 month after discharge, all steroids were completely discontinued. Follow-up for 7 months to date has shown no recurrence of acute joint pain. The patient reports no impact on diet, sleep, or daily activities, follows medical advice, remains physically active, consumes a low-purine diet, and continues urate-lowering therapy with febuxostat at a dose of 40 mg/day. Detailed changes in the patient’s main laboratory parameters are shown in Figure 3.

## Discussion

Gout is a common metabolic disorder characterized by elevated levels of uric acid, leading to the deposition of monosodium urate crystals in joints and other organs, including the kidneys. The inflammatory response mediated by macrophages and neutrophils towards monosodium urate crystal leads to acute gout, which can progress to chronic refractory gout (2). Chronic refractory gout typically manifests as recurrent gout attacks, making its treatment a significant challenge. Due to the chronic nature of the disease, most patients have already received treatment with gout-related medications, often with suboptimal efficacy. In clinical practice, there are many limitations in using medications during acute gout episodes. For example, the therapeutic dosage of colchicine is close to its toxic dosage. Asian populations, who generally have smaller physiques, are more prone to experiencing adverse drug reactions. In addition to the gastrointestinal reactions and liver or kidney functional impairments caused by the drugs themselves, caution should also be exercised regarding drug interactions. For instance, co-administration of colchicine with statins has been reported to increase the risk of rhabdomyolysis (3–5). In the present case, the patient had impaired renal function, but after adjustment of the medication regimen, their creatinine levels returned to normal.

The difficulty in medication selection for refractory gout has been a hot topic of research, as the long-term and recurrent nature of the disease poses significant challenges in patient treatment. Nonsteroidal anti-inflammatory drugs (NSAIDs) are currently among the most commonly prescribed and over-the-counter medications used for gout. However, their misuse or noncompliance by gout patients often leads to numerous adverse reactions such as gastrointestinal bleeding, liver and kidney damage, and cardiovascular toxicity, making the treatment of chronic refractory gout even more challenging (6, 7). Glucocorticoids are not recommended as first-line treatment for chronic refractory gout. They are advised to be used only in patients with severe systemic symptoms when NSAIDs are ineffective, or in elderly patients, those with impaired liver or kidney function, or heart failure. However, glucocorticoids also carry risks of serious adverse reactions such as increased infection susceptibility, gastrointestinal ulcers and bleeding, and osteoporosis.

In recent years, biologic agents have gained a place in the treatment of gouty arthritis. There have been case reports on the use of tumor necrosis factor-alpha antagonists, such as etanercept, for the treatment of refractory gout since 2004 (8). IL-6 inhibitors have also been mentioned in the treatment regimens for refractory gout (9, 10). IL-1 receptor antagonists, as a novel therapeutic approach, have been selectively recommended in guidelines for the treatment of gouty arthritis that is poorly tolerant to the above-mentioned therapies or has contraindications. However, these drugs are expensive and not widely used in clinical practice due to limited availability in China. In this case, the patient received all medications except for IL-1 receptor antagonists, including some common biologic agents (IL-6 inhibitors and tumor necrosis factor-alpha antagonists), but the results were still unsatisfactory.

Monosodium urate (MSU) crystal deposition is the pathogenic factor in gout. Multiple studies have shown that MSU crystals can activate various immune cells to release interleukin-1β (IL-1β), tumor necrosis factor-alpha, interleukin-6, and other inflammatory mediators (11). Upadacitinib (UPA) is a novel selective JAK inhibitor designed to provide more potent and selective inhibition of JAK1 while reducing the impact on the physiological functions of other JAK subunits (such as hematopoiesis and immune function) (12, 13). Further cell analysis has demonstrated that UPA inhibits JAK1-dependent cytokines such as IL-6, IL-2, and IFN-γ with approximately 60 times the potency compared to erythropoietin signaling (mediated by JAK2) (14). As a new selective JAK inhibitor, UPA exhibits higher selectivity for JAK1 and potential more significant inhibitory effects than other subtypes.

Currently, UPA has been studied in the field of rheumatic diseases, specifically rheumatoid arthritis (RA). From 2015 to 2019, six multicenter (15–20), randomized, double-blind clinical trials were conducted with UPA 15 and 30 mg/day in patients with moderate to severe active RA, who had either not received prior treatment with methotrexate (MTX) or showed inadequate response (IR) to MTX or biological disease-modifying antirheumatic drugs (bDMARDs). The results showed effective responses in these patient populations, and the safety data were similar to MTX or adalimumab (ADA). In 2019, the European League Against Rheumatism (EULAR) updated their treatment recommendations for RA, recommending JAK inhibitors as equivalent to biologics. In 2021, the US FDA modified the indication for UPA as a treatment for adults with moderate to severe active RA who have had an inadequate response or intolerance to one or more TNF inhibitors (TNFi). In China, UPA was approved in 2022 for the treatment of adult patients with moderate to severe active RA who have had an inadequate response or intolerance to one or more TNFi. Research has also been conducted on related diseases such as psoriatic arthritis (PsA) and ankylosing spondylitis (AS) (21–25). However, to the best of our knowledge, there have been no reported cases of using UPA for the treatment of gouty arthritis.

We present a case of a typical patient with chronic refractory gout, characterized by a prolonged course of the disease and a lack of appropriate treatment. During this acute episode, conventional treatments, though recognized for acute phase management, proved to be suboptimal, with high-dose steroids causing significant distress to the patient. After thorough discussion with the patient, a decision was made to supplement the treatment with upadacitinib. The outcome was remarkably positive, with successful tapering of steroids, improvement in joint pain, and a smooth discharge enabling the patient to resume normal activities. Subsequent follow-up revealed that after a two-month course of upadacitinib tablets, the patient was able to discontinue the medication while maintaining uric acid-lowering therapy. To the best of our knowledge, there are currently no documented cases demonstrating the successful treatment of refractory gout with upadacitinib extended-release tablets. Therefore, our understanding of the related mechanisms is limited. However, based on our considerations, the mechanism by which upadacitinib alleviates acute gout attacks may be related to the following aspects: (1) inhibition of cytokine signaling pathways, such as IL-6, IL-2, IL-12, and IFN-γ, as inflammatory factors play a crucial role in gout attacks (26, 27). (2) Regulation of regulatory T cells: the inhibitory effect of upadacitinib also involves the function of regulatory T cells (Treg). Upadacitinib can increase the number and function of Treg cells, thereby mitigating excessive immune system activation through immune response regulation (28). Research has indicated that the dynamic evolution of Treg cells, Th17 cells, and other immune cells is closely related to the pathogenesis of gout and the inflammatory response, primarily manifested in T cell-mediated cellular immunity and B cell-mediated humoral immune responses (29). In addition, upadacitinib can inhibit the migration of inflammatory cells. The aggregation and migration of inflammatory cells are crucial features of the inflammatory response, and upadacitinib can inhibit the production and release of relevant signaling molecules in inflammatory cells, thereby reducing their aggregation and migration at the site of inflammation (30). However, these findings are based on individual cases, and further research with more cases and experiments is needed to confirm the specific mechanisms and reasons. This case study serves as a reference point. Regarding the safety of this medication, we have been monitoring it closely. Since the patient only used the medication for 2 months to manage the acute phase, which is a relatively short duration, serious potential side effects of the drug include major cardiovascular events, cancer, thrombosis, and the risk of death (31), as well as common skin rashes (32, 33), None of these adverse effects were observed during the follow-up period in this case.

## Conclusion

Upadacitinib tablets may have a certain therapeutic effect on acute attacks of chronic refractory gouty arthritis.

## Data availability statement

The original contributions presented in the study are included in the article/supplementary material, further inquiries can be directed to the corresponding author.

## Ethics statement

The studies involving humans were approved by Ethics Committee of Lishui Central Hospital. The studies were conducted in accordance with the local legislation and institutional requirements. The participants provided their written informed consent to participate in this study. Written informed consent was obtained from the individual(s) for the publication of any potentially identifiable images or data included in this article.

## Author contributions

LY: Writing – original draft. WH: Writing – review & editing. HL: Writing – review & editing. LJ: Writing – review & editing. WL: Writing – review & editing.
